# Accurate Real-Time PCR Strategy for Monitoring Bloodstream Parasitic Loads in Chagas Disease Patients

**DOI:** 10.1371/journal.pntd.0000419

**Published:** 2009-04-21

**Authors:** Tomas Duffy, Margarita Bisio, Jaime Altcheh, Juan Miguel Burgos, Mirta Diez, Mariano Jorge Levin, Roberto Rene Favaloro, Hector Freilij, Alejandro Gabriel Schijman

**Affiliations:** 1 Laboratorio de Biología Molecular de la Enfermedad de Chagas, Instituto de Investigaciones en Ingeniería Genética y Biología Molecular (INGEBI-CONICET), Buenos Aires, Argentina; 2 Parasitology Unit of the “Ricardo Gutierrez” Children's Hospital, Buenos Aires, Argentina; 3 Transplant Unit of the Instituto de Cardiología y Cirugía Cardiovascular, Fundación “René Favaloro”, Buenos Aires, Argentina; New York University School of Medicine, United States of America

## Abstract

**Background:**

This report describes a real-time PCR (Q-PCR) strategy to quantify *Trypanosoma cruzi* (*T. cruzi*) DNA in peripheral blood samples from Chagas disease patients targeted to conserved motifs within the repetitive satellite sequence.

**Methodology/Principal Findings:**

The Q-PCR has a detection limit of 0.1 and 0.01 parasites/mL, with a dynamic range of 10^6^ and 10^7^ for Silvio X10 cl1 (*T. cruzi* I) and Cl Brener stocks (*T. cruzi* IIe), respectively, an efficiency of 99%, and a coefficient of determination (*R* ^2^) of 0.998. In order to express accurately the parasitic loads: (1) we adapted a commercial kit based on silica-membrane technology to enable efficient processing of Guanidine Hydrochloride-EDTA treated blood samples and minimize PCR inhibition; (2) results were normalized incorporating a linearized plasmid as an internal standard of the whole procedure; and (3) a correction factor according to the representativity of satellite sequences in each parasite lineage group was determined using a modified real-time PCR protocol (Lg-PCR). The Q-PCR strategy was applied (1) to estimate basal parasite loads in 43 pediatric Chagas disease patients, (2) to follow-up 38 of them receiving treatment with benznidazole, and (3) to monitor three chronic Chagas heart disease patients who underwent heart-transplantation and displayed events of clinical reactivation due to immunosupression.

**Conclusion/Significance:**

All together, the high analytical sensitivity of the Q-PCR strategy, the low levels of intra- and inter-assay variations, as well as the accuracy provided by the Lg-PCR based correction factor support this methodology as a key laboratory tool for monitoring clinical reactivation and etiological treatment outcome in Chagas disease patients.

## Author Summary

Infection with the parasite *Trypanosoma cruzi* (*T. cruzi*), causing American trypanosomiasis or Chagas disease, remains a major public health concern in 21 endemic countries of America, with an estimated prevalence of 8 million infected people. Chagas disease shows a variable clinical course, ranging from asymptomatic to chronic stages with low parasitaemias, whose severest form is heart disease. Diagnosis at the asymptomatic and chronic stages is based on serological detection of anti-*T. cruzi* antibodies, because conventional parasitological methods lack sensitivity. Current chemotherapies are more effective in recent infections than in the chronic adult population. The criterion of cure relies on serological conversion to negative, which may occur only years after treatment, requiring long-term follow-up. In this context, we aimed to develop a real-time PCR assay targeted to repetitive sequences of *T. cruzi* for sensitive quantitation of parasitic load in peripheral blood of infected patients. It was applied to monitor treatment response of infected children, allowing rapid evaluation of drug efficacy as well as detection of treatment failure. It was also used for early diagnosis of chagasic reactivation in end-stage heart disease patients who received immunosuppressive drugs after cardiac transplantation. This laboratory strategy may constitute a novel parasitological tool for prompt and sensitive evaluation of anti-parasitic treatment of Chagas disease.

## Introduction

Infection with the parasite *Trypanosoma cruzi* (*T. cruzi*) remains a major concern in 21 endemic countries of America, with an estimated prevalence of almost 8 million infected people [1]. The infection may be acquired mainly through the triatomid insect vector, blood transfusion or the trans-placental route. Furthermore, in areas under vector control, cases of congenital and transfusional transmission are relatively emerging [2]. This parasitic disease shows a variable clinical course, which ranges from asymptomatic cases, to severe chronic stages characterized by low parasitaemia and cardiac and/or gastrointestinal disorders [1].

Individuals from different endemic regions are infected with distinct parasite populations that may play a role in pathogenesis, clinical forms and severity of the disease [3]. Parasite populations are classified into two main phylogenetic lineages, *T. cruzi* I (TcI) and *T. cruzi* II (TcII) [4]; the later composed by five subdivisions designated as TcIIa to TcIIe [5].

Current chemotherapies based on the nitrofuran nifurtimox, and the nitroimidazole benznidazole, are unsatisfactory since these compounds are almost exclusively effective in recent infections and frequently have toxic side effects [6]. In this context, the development of novel drugs is necessary [6].

After etiological treatment (tmt), the criterion of cure relies on serological conversion to negative of the anti- *T. cruzi* antibody response [2], but in patients initiating therapy at the indeterminate phase, seroconversion usually occurs several years after treatment, requiring long-term follow-up [7]. Moreover, parasitological response to treatment is usually monitored by means of traditional methods such as Strout, hemoculture or xenodiagnoses, which lack sensitivity, and therefore are also inadequate for these purposes [2]. In this context, quantitative real-time PCR (Q-PCR) has the potential to become a novel parasitological tool for prompt evaluation of trypanocidal treatment. As a target for amplification, the nuclear satellite DNA, represented in 10^4^ to 10^5^ copies in the parasite genome is highly conserved [8]–[11] and therefore may provide accurate Q-PCR based measurements. Proper Q-PCR performance also requires high quality DNA extraction procedures from blood samples, which in most cases are collected in guanidine hydrochloride and EDTA buffer (GEB) [12]. The co-purification of trace PCR inhibitors may not impede amplification but may reduce its efficiency resulting in erroneous quantification of the parasitic load.

Accordingly, we aimed to develop a satellite-DNA based Q-PCR strategy for accurate quantification of *T. cruzi* loads in peripheral blood samples along with an adequate DNA extraction protocol. The following features have been regarded:

1. A commercial DNA purification protocol based on silica-membrane technology was adapted for GEB samples, providing DNA lysates without PCR interfering substances.
2. A heterologous internal standard (IS) was incorporated to each GEB sample to follow-up the yield and quality of DNA extraction and PCR amplification.
3. The relative copy number of satellite repeats per genome, according to the parasite lineage, was assessed for a more accurate quantification of the parasitic load, by means of a real-time PCR melting curve analysis (Lg-PCR).

Finally, we applied this Q-PCR strategy to: (1) calculate the basal *T .cruzi* loads in blood specimens collected from Chagas disease pediatric patients, (2) follow-up their parasitological response to treatment with benznidazole, and (3) monitor *T. cruzi* recrudescence and parasitological response to treatment in chronic Chagas heart disease patients undergoing heart-transplantation and receiving immunosuppressive therapy.

## Materials and Methods

### Ethics statement

This study was conducted according to the principles expressed in the Declaration of Helsinki. The study was approved by the Institutional Review Boards of the “Ricardo Gutierrez” Children's Hospital and of the Fundacion “Rene Favaloro”. All patients or responsible adults provided written informed consent for the collection of samples and subsequent analysis.

### Parasite stocks

*T. cruzi* epimastigotes were grown in liver infusion tryptose (LIT) medium containing 10% calf serum at 27–28°C. The parasites were harvested and stored at −70°C. *T. cruzi* DNA was purified after Phenol-Chloroform extraction and ethanol precipitation.

Reference *T. cruzi* stocks used as controls were: TcI (Silvio X10 cl1, SN3, HA, Pal V2-2, Pav 00, G); TcIIa (CanIII); TcIIb: (Tu18, JG, Gilmar, Y, Basileu, Mas cl1); TcIIc: (M5631, Cu-Tom-229, Cu-Yaya-211), TcIId (Mn cl2, Bug 2148 cl1, SO3 cl5, PAH 265, Tev 41), TcIIe (Cl Brener, Tul 77, Tep 6, Tep 7, MC). They were kindly provided by Patricio Diosque (Universidad Nacional de Salta, Salta, Argentina), Andrea M. Macedo (University Federal of Minas Gerais, Belo Horizonte, Brasil), and Michel Tibayrenc (UR62 “Genetics of Infectious Diseases”, IRD Centre, Montpellier, France). Some *T. cruzi* I strains were provided by Omar Triana Chavez (University of Antioquia, Medellín, Colombia). Argentinean *T. cruzi* IIc isolates were provided by Ricardo Gurtler (Universidad de Buenos Aires, Argentina).

### Chagas disease patients

1. Peripheral blood samples collected from a cohort of 43 *T. cruzi*-seropositive pediatric patients (mean age: 7.13 years; 15 days–18 years old) admitted between 2005 and 2006 at the Parasitology Unit of the “Ricardo Gutierrez” Children's Hospital, a reference centre for diagnosis and treatment of Chagas disease pediatric patients (Government of Buenos Aires, Argentina). These patients were treated during 60 days with benznidazole (5 to 8 mg/kg/day) [13]. Parasitic loads were determined by Q-PCR at time of diagnosis (t1), at 7 (t2), 30 (t3) and 60 (t4) days of treatment. After t4, patients were followed-up by qualitative kDNA-PCR at 6, 12 and 18 months post-tmt.
2. Blood samples from three chronic Chagas heart disease patients undergoing orthotopic heart transplantation (Tx) in 2003–2005 were provided by the Transplant Unit of the Instituto de Cardiología y Cirugía Cardiovascular, Fundación “René Favaloro”, Buenos Aires, Argentina. [14].

All these protocols were approved by the ethical committees of the corresponding institutions and written informed consents were required from each patient or a responsible adult.

### DNA extraction from peripheral human blood samples

Ten mL or 2 mL blood samples collected from *T. cruzi* infected adults or infants, respectively, were immediately mixed with one volume of 2× lysis buffer containing 6 M guanidine hydrochloride (Sigma, St Louis, USA) and 200 mM EDTA, pH 8.0 (GE) [12]. QIAmp DNA Mini Kit (Qiagen, Valencia, CA) based extraction was carried out from 400 µl of GEB and eluted with 200 µl of water according to the manufacturer's instructions using the Blood and Body Fluid Spin Protocol, with slight modifications. Briefly, since blood samples were initially mixed with one volume of GE lysis buffer, treatment with proteinase K and “AL” lysis buffer (which contains guanidine hydrocloride) were omitted. The following steps were carried out following the manufacturer's instructions.

Phenol-chloroform based DNA extraction was carried out from 100 µl of GEB aliquots and resuspended in 50 µl water as reported [15].

### *T. cruzi* standard calibration curve

Cl Brener epimastigotes were added to non-infected human blood to result in a concentration of 10^5^ p/mL of reconstituted blood and immediately mixed with one volume of 2× lysis GE buffer. The resulting GEB was serially diluted 10-fold with non-infected GEB to cover a range between 10^5^ and 0.001 parasite equivalents/mL. Total DNA was purified using the QiAmp DNA Mini Kit based extraction method, as above described.

### *T. cruzi* satellite DNA Q-PCR

An MJR-Opticon II device (Promega, USA) was used for amplification and detection. The 20 µL reaction tube contained 0.5 µM of novel primers Sat Fw (5′-GCAGTCGGCKGATCGTTTTCG-3′) and Sat Rv (5′-TTCAGRGTTGTTTGGTGTCCAGTG-3′), 3 mM MgCl_2_, 250 µM of each dNTP, 0.5 U of Platinum Taq polymerase, (Invitrogen, Life Technologies, USA) SYBR Green (Invitrogen, Life Technologies, USA) at a final concentration of 0.5× and 2 µL of sample DNA. After 5 min of pre-incubation at 95°C, PCR amplification was carried out for 40 cycles (94°C for 10 s, 65°C for 10 s and 72°C for 10 s). The plate was read at 72°C at the end of each cycle.

### Internal standards of Q-PCR

A linearized p-ZErO plasmid containing a sequence of *Arabidopsis thaliana* was used as a heterologous internal standard (IS). All clinical samples were co-extracted with 200 pg of recombinant plasmid, which was assumed as 1 arbitrary unit (AU) of IS. This amount of IS input was chosen because the amplicons are detected at approximately the Cycle threshold (Ct) number 20, the mid point of the dynamic range of the PCR. For each Q-PCR test, the IS was added to 400 µl of GEB lysate, immediately before the DNA extraction procedure.

The standard calibration curve for the IS was carried out using the same reconstituted blood samples as for the *T. cruzi* calibration curve: those samples containing 10^5^, 10^4^ and 10^3^ p/mL were spiked with 2 AU of IS, those containing 100, 10 and 1 p/mL were spiked with 0.2 AU of IS, and those containing 0.1, 0.01 and 0.001 p/mL were spiked with 0.02 AU of IS. Two non-infected blood samples with and without IS DNA were used as negative controls of the extraction procedure.

The IS was quantified using 1 µM of primers, IS Fw (5′-AACCGTCATG GAACAGCACGTAC-3′) and IS Rv (5′-CTAGAACATTGGCTCCCGCAACA-3′). All other PCR reagents and cycling conditions were identical to those used for *T. cruzi* Q-PCR.

### Qualitative PCR detection of *T. cruzi* kDNA

Post-treatment follow-up of parasitological response to benznidazole in pediatric and heart transplanted patients was conducted by means of kDNA-PCR as previously reported [14],[16].

### Determination of the satellite/P2α ratio

In order to analyze the variability in the number of satellite sequences detected by Q-PCR, comparative quantification was performed using as a normalizer the single copy ribosomal protein P2α gene (GenBank accession number XM_800089).

Assays for quantification of P2α gene were performed using 1 µM of primers P2α Fw (5′-ATGTCCATGAAGTACCTCGCC-3′) and P2α Rv (5′-GCGAATTCTTACGCGCCCTCCGCCACG-3′). All other PCR reagents were used at the same concentrations as for *T. cruzi* Q-PCR. After 5 min of pre-incubation at 95°C, PCR amplification was carried out for 40 cycles (94°C for 10 s, 60°C for 15 s and 72°C for 10 s). The plate was read at 72°C at the end of each cycle.

### Identification of parasites according to the number of satellite sequences detected per genome by Lg-PCR

Since TcI and TcIIa parasites have a lower number of satellite sequences than TcIIb/c/d/e parasites, we have developed a method to distinguish between both groups according to the melting temperatures (Tm) of their corresponding amplicons to enable more precise parasitic load assessments.

The identification of the type of satellite sequence was performed using 0.5 µM of primers TcZ1 (5′-CGAGCTCTTGCCCACACGGGTGCT-3′) and Sat Rv (5′-TTCAGRGTTGTTTGGTGTCC AGTG-3′). All other PCR reagents were used at the same concentrations as for *T. cruzi* Q-PCR. The PCR conditions consisted of an initial denaturation at 95°C for 5 min, followed by 40 cycles of 94°C for 10 s, 65°C for 10 s and 72°C for 10 s with fluorescence acquisition at 81.5°C and a final step of 2 min at 72°C. Amplification was immediately followed by a melt program with an initial denaturation of 5 s at 95°C and then a stepwise temperature increase of 0.1°C /s from 72–90°C.

Since satellite DNA is arranged in tandem repeats, if more than 0.05 parasites (equivalent to approximately 10 p/mL) are loaded in the reaction tube, a satellite sequence dimer is amplified giving place to a melting temperature peak typically above 86°C for both lineage groups.

Satellite sequences were obtained by direct sequencing of satellite DNA amplicons obtained with TcZ1 and TcZ2 primers (GenBank Accession numbers EU728662-EU728667). Sequence alignment was conducted using MEGA version 4 [17].

### Normalization of parasite loads according to the internal standard and parasite satellite sequence group

The parasite load in the clinical sample was normalized respect to the standard curve according to (1) the efficiency of the DNA extraction procedure measured by the amplification of the IS, and (2) the parasite lineage group. The following equation was used: *N_p/mL_* = (*N_p/well_*×*LF* / *AU*) / *V*, where *N_p/mL_* is the number of parasites per millilitre of blood, *N_p/well_* is the number of parasites per well, *LF* is the lineage factor, *AU* are the arbitrary units of IS quantified and *V* is the volume of extracted DNA sample used per reaction.

## Results

### Analytical sensitivity and reproducibility of Q-PCR with purified DNA and reconstituted blood samples

**Figure 1:**
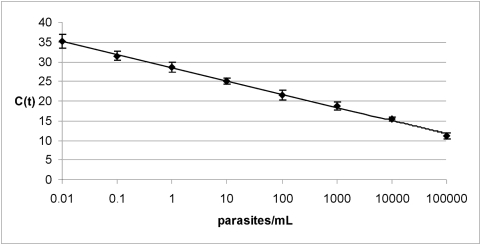
Dynamic range of the *T. cruzi* satellite DNA based Q-PCR. Results are expressed as the number of parasites per milliliter of blood and represent the average of 5 independent experiments. Slope = −3.35. Efficiency = 99%. Dynamic range: 0.01–10^5^ p/mL. *R* square: 0.998. C(t): cycle threshold.

The analytical sensitivity of the Q-PCR was tested by using serial dilutions of purified *T. cruzi* DNAs from TcI (Silvio X10 cl1) and TcIIe (Cl Brener) reference stocks. The detection limits were 2 fg and 0.2 fg DNA per reaction tube with a dynamic range of 10^7^ and 10^8^ for Silvio X10 cl1 and Cl Brener stocks, respectively (data not shown). These detection limits correspond to 0.01 and 0.001 parasite genomic equivalents considering that one parasite cell harbors approximately 200 fg of DNA. Furthermore, we tested the operational parameters of Q-PCR in reconstituted - blood samples spiked with known quantities of Cl Brener and Silvio X10 cl1 cultured epimastigote cells. The dynamic range of Q-PCR performed with samples reconstituted with Silvio X-10 was 0.1–10^5^ p/mL and with those spiked with Cl Brener was 0.01–10^5^ p/mL (Figure 1).

The reproducibility of the Q-PCR assay at 100 p/mL and 1 p/mL was estimated by testing each reconstituted sample 15 times in the same PCR run. The coefficients of variation of the Ct values were 1.27% and 2.30%, respectively for Cl Brener and 1.60% and 5.42%, respectively for Silvio X10 cl1.

The reproducibility of the DNA extraction was also characterised: aliquots from the same GEB-reconstituted samples containing 10 Cl Brener p/mL were processed in twenty independent DNA purification experiments. For each of the 20 DNA lysates, the p/mL were measured in triplicate PCR runs and a mean value was calculated. The coefficient of variation of the Ct values among the 20 mean values was 1.69%.

### Estimation of the relative numbers of satellite DNA copies per genome for different *Trypanosoma cruzi* lineages

In order to evaluate if the differences in the detection limits of the Q-PCR obtained from Cl Brener or Silvio X10 cl1 samples were related to a different copy number of satellite repeats in their respective genomes, the amounts of satellite sequences, relative to the single copy gene encoding the ribosomal-P2α protein, were estimated for parasite stocks belonging to the 6 lineages (Table 1). The stocks belonging to TcIIb/d/e lineages showed similar amounts of satellite repeats (Table 1A), the M5631 stock (TcIIc) harbored 2-fold less repeats than the aforementioned stocks (Table 1A), whereas Can III stock (TcIIa) and six stocks belonging to *T. cruzi* I from Argentina, Colombia and Brazil, harbored a 10-fold lower number of satellite repeats (Table 1A and 1B). This is in agreement with the analytical sensitivities obtained with purified DNA and reconstituted samples of the reference stocks.

**Table 1:** Variation in the relative numbers of satellite DNA copies per genome.

A. Cultured *T. cruzi* stocks representing each of the phylogenetic lineages		
Lineage	*T.cruzi* Reference Stocks	Sat/P2α
**Tc I**	Silvio X-10 cl1	0.08
**Tc IIa**	Can lll	0.08
**Tc IIb**	Tu18	0.95
**Tc IIc**	M5631	0.47
**Tc IId**	Mn cl2	1.1
**Tc IIe**	Cl Brener	1

B. *T. cruzi* I stocks from different endemic countries		
*T. cruzi* I Stocks	Country of Origin	Sat/P2a
**Silvio X-10 cl1**	Brasil	0.08
**G**	Brasil	0,13
**HA**	Colombia	0,11
**SN3**	Colombia	0,13
**Pav 00**	Argentina	0,09
**Pal V2-2**	Argentina	0,12

Results are expressed as arbitrary units, obtained calculating the ratio of copy numbers of Cl Brener satellite/ ribosomal-P2α gene DNA targets.

### Distinction by Lg-PCR of *T. cruzi* groups according to the relative number of satellite repeats

**Figure 2:**
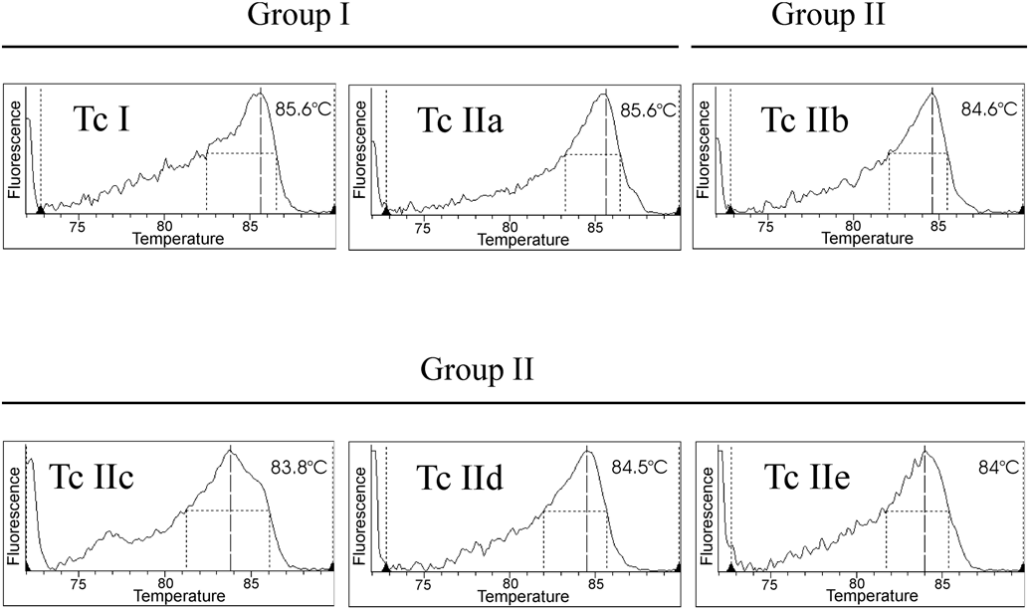
Melting curve analysis of satellite amplicons from reference stocks. Group I satellite amplicons show melting temperatures above 85°C, whereas Group II render amplification products with melting temperatures below 85°C.

Two groups of lineages, *T. cruzi* I/IIa (group I) and *T. cruzi* II (IIb,c,d,e) (group II) were clearly distinguished due to the differential melting temperatures of their corresponding satellite sequence amplicons, above or below 85°C, respectively (Figure 2).

The Lg-PCR was validated using a panel of 25 characterised stocks (Table 2): five stocks belonging to TcI, one to TcIIa, six to TcIIb, three to TcIIc, five to TcIId and the remaining five to TcIIe.

**Table 2:** Melting temperatures of satellite fragments amplified from *T. cruzi* stocks belonging to the 6 phylogenetic lineages.

Stock	Tm (°C)	Lineage	Tm (°C)±SD
Silvio X-10 cl1	85.7	Tc I	85.34±0.15
Pal v2-2	85.4		
G	85.5		
SN3	85.3		
HA	85.4		
Can lll	85.7	Tc lla	85.70±0.30
Tu18	84.6	Tc llb	84.60±0.18
JG	84.8		
Gilmar	84.8		
Y	84.6		
Basileu	84.4		
Mas cl1	84.4		
M5631	83.9	Tc llc	84.03±0.45
Cu-TOM-229	84.5		
Cu-Yaya-211	83.6		
Mn cl2	84.4	Tc lld	84.44±0.30
Bug 2148 cl1	84.8		
SO3 cl5	84.7		
PAH265	84.1		
Tev 41	84.2		
Cl Brener	84.2	Tc lle	84.42±0.33
Tul77	84.7		
Tep7	84.3		
MC	84.0		
Tep6	84.9		

The 99% confidence interval of the melting temperatures depicted by the *T.cruzi* strains from group I (mean: 85.5°C; CI 99% 85.2–85.8) and II (mean: 84.4°C; CI 99% 84.2–84.7) did not overlap. This distinction allows a more accurate parasite load quantification by incorporating a correction factor (Lineage Factor *LF*), according to the number of target sequences, when the parasitic load is calculated.

### Comparison of PCR inhibition using two extraction methods in human samples

We extracted 55 GEB samples from Chagas disease patients with the QIAmp DNA Mini Kit adapted protocol as well as with a Ph-Chl based method. Using the Ph-Chl based method, we detected traces of PCR inhibitors in 16 (29%) samples, whereas for the commercial kit we did not detect PCR inhibitors in any of the samples. The presence of PCR inhibitors was assessed by (1) the low yield of IS amplification, giving rise to less than 0.1 AU of the IS and (2) the improved yield of IS amplification when the DNA lysate was diluted 1/100 prior to the Q-PCR run.

Out of the 16 samples that presented traces of PCR inhibitors when extracted with the Ph-Chl based method, 4 were negative for *T. cruzi* by both extraction methods, 3 rendered similar parasite loads by both methods, 8 rendered higher parasite loads when extracted with the commercial kit, and one rendered a positive result only when extracted with the commercial kit. For the 39 samples extracted by Ph:Chl that did not contain PCR inhibitors, similar results of *T. cruzi* quantification were obtained with both methods of extraction. On the basis of these data, the QIAmp DNA Mini Kit extraction protocol was selected for Q-PCR in clinical samples.

### Application of the Q-PCR assay to clinical specimens

A panel of human blood samples was analyzed using the Q-PCR strategy, namely samples from seropositive pediatric patients and chronic Chagas heart disease adults presenting clinical reactivation after heart transplantation.

Table 3 describes the necessary steps for calculating the parasitic loads, considering the lineage factor (LF), the AU of the IS and the input volume of DNA sample. For example, in case Tx1c, the Q-PCR alone quantified 0.39 p/mL, but the AU of the IS was 0.43, and the LF was 10 (*T. cruzi* I), giving a final result of 9.07 p/mL, which is 23 times higher than if the quantification had been made based only on the Q-PCR crude measurement. In case Pd6, a pediatric patient with very low parasitemia, the lineage could not be determined since the low concentration of amplicons gave rise to non-reliable melting temperature peaks and therefore the parasitic load could only be expressed within a range of 10-fold. In cases Tx1a and Tx2c, also with low levels of parasitemia, the lineage was presumed from previous data obtained from genotyped samples of the same patients, because it was demonstrated that the *T. cruzi* lineages were persistent during recrudescence leading to clinical reactivation in these patients [14]. Cases Pd2a and Tx2b presented high parasitic loads, 512 and 468 p/mL, respectively. In both of them, dimers of the satellite repeats were preferentially amplified when Lg-PCR was performed, giving melting temperatures above 86°C (Table 3). Thus, when the non-corrected Q-PCR results are above 10 p/mL, the DNA lysate should be diluted to approximately 1 p/mL before performing Lg-PCR.

**Table 3:** Examples of the calculation of parasitic loads in pediatric and transplanted patients.

Case	Age/Gender	Clinical Diagnosis	Sample	Q-PCR *N_p/well_ / V*	IS-PCR *AU*	Lg-PCR Tm (°C)	Lineage	*LF*	Parasitic Load *N_p/mL_* = (*N_p/well_*×*LF* / *AU*) / *V*
Pd 1a	7 y / M	Congenital ChD	Pre-tmt	7.20	0.87	84.6	2	1	8.28
1b			7 days of tmt	0.1	0.97	84.6	2	1	0.1
1c			30 days of tmt	0.76	1.21	84.4	2	1	0.63
1d			60 days of tmt	0.95	0.82	84.6	2	1	1.16
Pd 2a	3 m / M	Congenital ChD	Pre-tmt	527	1.03	84.5‡	2	1	512
2b			30 days of tmt	0.29	0.67	84.8	2	1	0.43
2c			60 days of tmt	1.01	1.34	84.4	2	1	0.75
Pd 3	1 y / F	Congenital ChD	Pre-tmt	14.69	1.27	84.4	2	1	11.57
Pd 4	2 y / M	Congenital ChD	Pre-tmt	0.34	0.49	84.6	2	1	0.69
Pd 5	3 y / F	Congenital ChD	Pre-tmt	0.54	1.18	84.6	2	1	0.46
Pd 6	10 y / F	Indeterminate ChD	Pre-tmt	0.04	0.74	ND	ND#	ND#	0.05–0.5
Tx 1a	46 y / M	Chronic Cardiomyopathy with Heart Transplantation	5 days pre-Tx	0.03	1.26	ND	1*	10*	0.22
1b			29 days post-Tx	0.59	1.53	85.2	1	10	3.85
1c			reactivation 78 days post-Tx	0.39	0.43	85.3	1	10	9.07
Tx 2a	61 y / F	Chronic Cardiomyopathy with Heart Transplantation	7 days post-Tx	2.23	0.84	84.5	2	1	2.66
2b			reactivation 92 days post-Tx	342	0.73	84.6‡	2	1	468
2c			12 days post-tmt	0.13	1.84	ND	2*	1*	0.07

In the samples from transplanted patients (Tx) whose infecting lineages could not be determined, they were presumed from the data obtained from other samples of the same patient, assuming that the lineage is persistent during reactivation [13]. ND: Not determined.

# In case Pd6 no presumption could be made about the parasite lineage.

**‡:** Samples diluted before Lg-PCR.

The reproducibility of the whole Q-PCR assay was evaluated in 5 clinical samples from different Chagas disease patients covering a range of 3 logarithms of parasitic loads (0.06 to 70.29 p/mL, Table 4). For each peripheral blood sample (A to E, Table 4), the entire protocol was carried out in 5 independent replicates. The coefficients of variation of the Ct values and p/mL for the *T.cruzi* Q-PCRs ranged from 1.30 to 5.49% and from 25.19 to 137,95%, respectively, and those for the IS-PCRs ranged from 0.66 to 2.28% and from 9.56 to 29.51%, respectively (Table 4). The final parasitic load measurements were inversely correlated with their coefficients of variation, which ranged from 32.43 to 114.20% (Table 4).

**Table 4:** Reproducibility of the Q-PCR and IS-PCR assays in clinical samples of Chagas disease patients.

Patient	Replicates	Q-PCR		Cv %		IS-PCR		Cv%		Parasitic Load		
		Ct	p/mL	Ct	p/mL	Ct	AU	Ct	AU	p/mL	Mean p/mL	Cv%
**A**	1	neg	0.00	5.49	137.95	20.83	1.37	1.54	22.66	0.00	0.06	114.2
	2	30.29	0.29			20.40	1.83			0.16		
	3	31.95	0.09			21.23	1.05			0.09		
	4	34.31	0.02			20.91	1.30			0.01		
	5	33.56	0.03			21.12	1.13			0.03		
**B**	6	30.83	0.20	2.83	66.52	23.08	0.30	2.28	29.51	0.65	0.33	72.39
	7	31,70	0.11			22.48	0.46			0.24		
	8	30.08	0.33			21.97	0.64			0.51		
	9	32.13	0.08			22.00	0.63			0.13		
	10	32.09	0.08			21.86	0.69			0.12		
**C**	11	30.16	0.31	3.20	69.45	21.64	0.80	1.51	21.96	0.39	0.50	88.15
	12	30.29	0.29			20.94	1.28			0.22		
	13	31.19	0.15			21.31	1.00			0.15		
	14	28.59	0.92			21.76	0.74			1.24		
	15	29.59	0.46			21.31	1.00			0.46		
**D**	16	26.08	5.16	1.30	25.19	20.97	1.25	0.66	9.56	4.13	4.87	32.43
	17	25.93	5.72			21.03	1.20			4.75		
	18	25.36	8.47			21.11	1.14			7.41		
	19	26.26	4.56			20.75	1.45			3.15		
	20	25.91	5.80			21.05	1.18			4,90		
**E**	21	22.89	46.25	2.44	40.68	21.36	0.96	1.42	19.05	47.99	70.29	33.45
	22	22,80	49.20			21.95	0.65			76.01		
	23	23,00	42.88			21.32	0.99			43.32		
	24	21.72	103.38			21.16	1.10			93.84		
	25	22.17	75.87			21.56	0.84			90.28		

The *T.cruzi* lineage group was assessed by Lg-PCR as *T.cruzi* II in all tested patients. Parasitic Loads were calculated as shown in Table 3.

### Follow-up of pediatric patients under treatment with benznidazole

**Figure 3:**
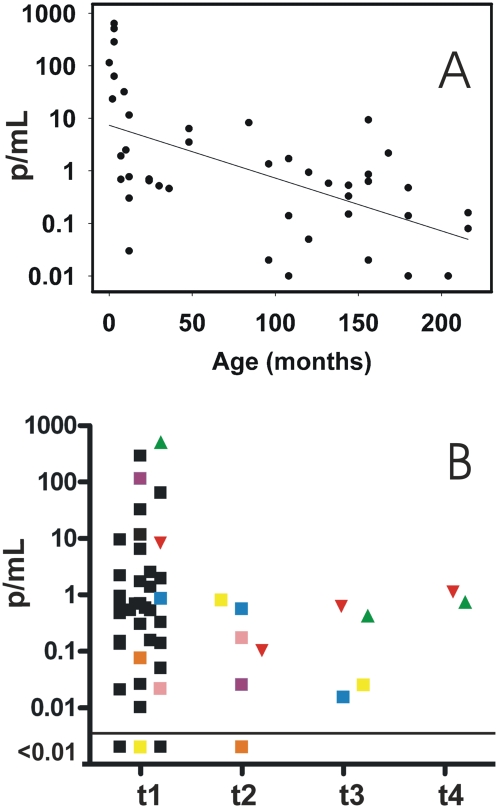
Parasitic loads in peripheral blood samples from pediatric patients. (A) Association between basal parasitic loads and patients' ages in 43 pediatric cases. Coefficient of correlation: −0.5832; P<0.05. (B) Monitoring of parasitological response to benznidazole therapy in 38 pediatric patients. The evolution of the parasitic loads for patients with more then one positive sample are depicted. Samples were withdrawn at time of diagnosis (t1), after 7 (t2) and 30 (t3) days of treatment, as well as at the end of treatment (t4, 60 days). Only the PCR positive samples are shown. The horizontal line represents the lower limit of the dynamic range of Q-PCR.

The pre-treatment parasitic loads were assessed in 43 children with Chagas disease. Basal parasitic loads ranged from 640 to 0.01 p/mL, and were correlated to the patients' ages at the time of diagnosis (coefficient of Spearman: −0.5832, P<0.05) (Figure 3A). Thirty eight of these patients were monitored by Q-PCR during 60 days of etiological treatment with benznidazole. Parasitic loads were determined at time of diagnosis (t1) in 38 cases, at 7 (t2), 30 (t3) and 60 (t4) days of treatment in 31 cases.

Figure 3B and Table 3 show the parasitic response of these patients during treatment monitoring. Q-PCR results at t2 were negative in 24 out of 31 patients (77%), at t3 in 27 out of 31 patients (87%) and at t4 in 29 out of 31 patients (94%).

One of the Q-PCR positive patients at t4 was a 7 year-old boy whose parasite load declined from 8.28 p/mL at t1 (Pd1a) to 0.1 p/mL at t2 (Pd1b), relapsing to 0.63 p/mL at t3 (Pd1c) and 1.16 p/mL at t4 (Pd1d) (Figure 3B, red triangle, Table 3).

The other Q-PCR positive patient at t4 was a 3 month old infant who displayed detectable parasitic loads in the three analysed samples; 512 p/mL at t1 (Pd2a), 0.43 p/mL at t3 (Pd2b) and 0.75 p/mL at t4 (Pd2c) (Figure 3B, green triangle, Table 3). Both patients were followed-up by kDNA-PCR with persistently positive results at 6, 12 and 18 months post-tmt suggesting treatment failure (data not shown).

### Q-PCR based monitoring of Chagas disease reactivation in heart-transplanted patients

**Figure 4:**
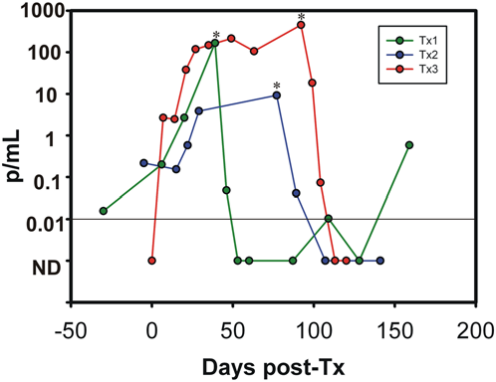
Follow-up of Chronic Chagas heart disease patients after heart transplantation. Parasitic loads in peripheral blood samples of Chronic Chagas heart disease patients with clinical reactivation due to immunosupression after heart transplantation. * Time of diagnosis of clinical reactivation and etiological treatment.

Stored peripheral blood samples from three heart transplanted patients infected with different parasite lineages and presenting different patterns of clinical reactivation were retrospectively analyzed using the Q-PCR strategy (Figure 4).

Case Tx1 presented positive Q-PCR results (0.22 p/mL) prior to heart transplantation. Clinical reactivation was diagnosed at day 78 post-Tx by a skin chagoma biopsy and a positive Strout result. The parasite population was characterized as group I by Lg-PCR.

Case Tx2 did not present positive PCR results before heart Tx, but parasitemia became detectable 7 days post-Tx (2.66 p/mL). Clinical reactivation was diagnosed at 92 days post-Tx due to positive Strout findings. Upon etiological tmt, the parasitic load decreased reaching undetectable levels at 21 days post-tmt. The *T. cruzi* population was identified as group II.

Case Tx3 presented a parasite load of 0.02 p/mL 30 days before Tx, showing a 10-fold increase (0.2 p/mL) 7 days after Tx. The parasite load continued to rise until clinical reactivation was diagnosed 38 days after Tx based on positive Strout findings. Accordingly, the patient was treated with benznidazole and the parasite load dropped rapidly, with negative Q-PCR findings at 14 days post-tmt, persisting PCR negative for 53 days. However, 70 days after tmt Q-PCR monitoring revealed detectable loads (0.01 p/mL) (Figure 4). The bloodstream *T. cruzi* population was identified as group II.

## Discussion

Herein we report a highly sensitive, reproducible, accurate and rapid real-time Q-PCR strategy for quantification of the *T. cruzi* parasitic load in human blood samples. Indeed, this is a first attempt to develop a real-time Q-PCR strategy for reliable *T. cruzi* quantification, because it incorporates: (1) a commercial kit for sample processing which minimizes carry-over of PCR inhibitors and standardizes the yield and quality of DNA extraction, (2) a closed-tube single-round PCR reaction which minimizes carry-over contamination, (3) an appropriate internal quality control and (4) a correction of the parasitic load, according to the variation in the number of target sequences between the different lineage groups. This is a key step towards the standardization and validation of in-house Q-PCR tests for application to routine laboratory practice.

Although studies showing the advantages of real-time PCR for screening and quantification of *T. cruzi* have recently appeared, none have implemented procedures to normalize the DNA extraction yield and the representativity of the PCR target according to the lineage group. Some studies [18]–[20] have chosen as an internal control a host DNA sequence, but the human DNA content in blood can be highly variable, especially in immunosupressed patients. In this work, the addition of a standardized amount of a plasmid containing a heterologous sequence, allows normalization of the DNA extraction yields and detection of false negatives due to inhibition under any clinical situation.

Regarding the selected molecular target of amplification, Elias et al. [8],[11] and Vargas et al. [10] demonstrated that satellite DNA is 4 to 9 times more abundant in TcIIb/d/e than in TcI stocks. Herein, we have extended this analysis to all 6 *T. cruzi* lineages, describing for the first time the satellite repeats of *T. cruzi* IIa, IIc and IId representative stocks (GenBank Accession numbers EU728662-EU728667). Indeed, we detected a 5 to 10-fold variation in the satellite DNA content between group I (TcI/IIa) and group II (TcIIb/c/d/e) parasite stocks (Table 1). Therefore, this variability must be taken into account in order to calculate accurately the parasitic loads. Accordingly, we have also designed a highly sensitive real-time PCR procedure (Lg-PCR) to distinguish *T. cruzi* group I (with lower satellite sequence copy number and higher melting temperature) from group II lineages (with higher satellite sequences copy number and lower melting temperature). All the analyzed stocks rendered only one temperature melting peak, including hybrid stocks like Cl Brener although harboring both types of satellite sequences. This can be explained by the fact that Cl Brener (Tc IIe) harbors ten times more type II than type I satellite repeats [10] and due to exponential amplification, only the predominant sequence type is detected. The high analytical sensitivity of Lg-PCR makes it useful for direct lineage group characterization in biological samples that may not be typed using other typing methods [16],[21],[22]. Alternatively, a recently reported multiplex PCR strategy might be useful for typing *T.cruzi* groups in clinical specimens [23] when a Q-PCR test targeted to satellite sequences is carried out.

Sample processing and DNA extraction must also be optimized for reliable quantification. In this direction, we adapted a commercial kit, based on silica-membrane technology (QIAmp DNA Mini Kit) for processing GEB samples. Phenol-chloroform DNA extraction is a cost-effective method for qualitative PCR, but traces of PCR inhibitors may be co-purified [24],[25]. These interfering substances may not impede Q-PCR amplification, but could affect its efficiency leading to inaccurate results. In fact, we detected traces of PCR inhibitors in 29% of the samples extracted with Ph-Chl. When present, these inhibitors underestimated the parasite loads in 67% of the positive samples, although they did not seriously affect the positivity of the PCR. These results suggest that Ph-Chl based extraction of GEB samples is not suitable for Q-PCR but can be used for qualitative purposes.

In this report, we applied the Q-PCR strategy to blood samples collected from patients under different clinical scenarios. Its wide dynamic range allowed direct measurements in cases with high parasitic loads such as immunosuppressed Chagas disease patients and congenitally infected newborns, as well as in cases with low parasitemias, such as patients at the indeterminate phase or under etiological treatment. In this sense, the coefficients of variation of the Q-PCR measurements obtained from clinical samples were similar to those obtained from reconstituted blood samples (Table 4). When applied to newborns, infants and children with *T. cruzi* infection, Q-PCR estimated their basal parasitic loads in a vast range, between 0.01 and 640 p/mL of blood (Figure 3A). The highest parasitic loads observed in the younger pediatric population are in agreement with the results obtained by conventional parasitological and kDNA-PCR analysis [15],[26]. Moreover, the lower parasitic loads detected in the older pediatric patients reflect their evolution to the indeterminate phase of congenital infection [15]. Furthermore, we were able to follow-up their parasitological responses to treatment with benznidazole (Figure 3B) with a favorable outcome in 94.7% (36/38) cases. It is worth to note that at t3, under 30 days of tmt, 4 patients still showed detectable parasitic loads. At the end of tmt (t4) 2 of them became Q-PCR negative and remained negative during 18 months of post-tmt follow-up. This observation emphasizes the importance of a treatment regimen of 60 days. Interviews with the mothers of the two patients who persisted Q-PCR positive at t4 revealed the non-adherence in one of them (Pd2, Table 3 and Figure 3B, green triangle), whereas in the other case (Pd1, Table 3 and Figure 3B, red triangle) persistence of parasitemia indicated lack of parasitological response to benznidazole. These cases demonstrate the usefulness of the Q-PCR assay as surrogate marker for early detection of treatment failure. Two pediatric patients (Figure 3B, pink and yellow squares) presented an increase of their parasitic loads from t1 to t2, showing a favorable parasitological response to treatment in the samples collected at t3 and t4, fact that was confirmed by means of kDNA-PCR in the successive post-tmt controls (data not shown). The lower parasitic loads detected at t1, before initiation of tmt, compared with t2, might be due to natural fluctuations of the parasitemia in chronic Chagas disease patients [27].

In this context, it is important to analyze serial blood samples to be able to observe an increasing or decreasing tendency in the parasitic loads in chronic patients under treatment.

The Q-PCR test was also used for early detection of *T. cruzi* reactivation after heart transplantation. This was visualized through the increment of the parasitic loads in all patients who presented clinical manifestations of reactivation [14]. In case Tx1, the patient is infected with *T. cruzi* I, and the number of parasites increased from 0.22 p/mL (5 days pre-Tx) to 9.07 p/mL (78 days post-Tx) when the patient presented signs and symptoms of skin reactivation and patent parasitemia [14]. In the other two tested cases, Tx2 and Tx3, who were infected with group II populations, the parasitic loads increments were notably higher (Table 3 and Figure 4). In Tx3, treatment with benznidazole after reactivation achieved transitory parasitological response because samples collected at 44 and 70 days after tmt were Q-PCR positive (Figure 4). Parasite relapse was confirmed by means of kDNA-PCR in successive samples until a second episode of clinical reactivation was diagnosed [14]. In Tx cases, the Q-PCR allowed to detect parasitic load increase, previous to diagnosis of reactivation, as well as to follow-up parasitological response during treatment with benznidazole.

All together, the high analytical sensitivity of the Q-PCR strategy, the low levels of intra- and inter-assay variation, as well as the accuracy provided by the Lg-PCR correction, promotes this method as a key laboratory tool to follow-up patients under etiological treatment or at risk of clinical reactivation. This will be of particular significance for future drug trials in which an early assessment of efficacy or failure is mandatory [28].
